# Effects of Mg-Palygorskite Modified Biochar on the Growth of *Sedum alfredii* Hance in Heavy Metal Contaminated Soil

**DOI:** 10.3390/plants14050790

**Published:** 2025-03-04

**Authors:** Tianwen Wang, Xianxiang Luo, Kun Chen, Hao Zheng, Zewei Song, Lize Zhao, Junhua Gong, Fengmin Li, Ruhai Liu

**Affiliations:** 1 Institute of Coastal Environmental Pollution Control, Ministry of Education Key Laboratory of Marine Environment and Ecology, Ocean University of China, Qingdao 266100, China; wangtianwen@stu.ouc.edu.cn (T.W.); qddxchenkun@163.com (K.C.); zhenghao2013@ouc.edu.cn (H.Z.); songzewei2022@163.com (Z.S.); zhaolize@stu.ouc.edu.cn (L.Z.); lifengmin@ouc.edu.cn (F.L.); ruhai@ouc.edu.cn (R.L.); 2 Sanya Oceanographic Institution, Ocean University of China, Sanya 572000, China; 3 Shikefeng Chemical Co., Ltd., Linyi 276024, China; gjh20011570@163.com

**Keywords:** soil heavy metals, modified biochar, soil remediation, *Sedum alfredii* Hance, adsorption

## Abstract

Heavy metal contamination of soil poses a serious threat to agricultural production and human health. Biochar modified with Mg and palygorskite can reduce the content of available heavy metals in soil; however, its passivation effect is affected by the modification method, and there is a lack of research on its impact on plant growth in heavily polluted soil. In this study, four types of modified biochar were prepared using MgCl_2_, palygorskite, and wood as raw materials, including MBC and MPB prepared by pre-modification and BCM and BPM prepared by post-modification. *Sedum alfredii* Hance was selected as the test plant, and a pot experiment was conducted to explore the effects of unmodified and modified biochar on the growth of *Sedum alfredii* Hance in heavily polluted soil with Cu, Pb, Zn, and Cd. Compared with the original biochar, the modified biochar, especially the pre-modified biochar, significantly increased the ash content, pH, O/C ratio, surface functional group count, and mineral content. The adsorption capacity for heavy metals was also significantly enhanced, with the main adsorption mechanisms being precipitation, complexation, and ion exchange. The four types of modified biochar promoted the growth and biomass of *Sedum alfredii* Hance to varying degrees, with the promotion effect in the order of MPB > MBC > BPM > BCM, and the effect was more significant with a 3% addition. The modified biochar significantly reduced the content of available heavy metals in the rhizosphere soil, with a passivation effect in the order of MPB > MBC > BPM > BCM, and the 3% addition had the greatest effect. Further analysis via the Mantel test and structural equation modeling confirmed that modified biochar promoted the growth of *Sedum alfredii* Hance by reducing the available heavy metal content in the rhizosphere soil and increasing the NO_3_^−^-N and AP contents. This study provides data support for the development of functionalized biochar for the remediation of heavy metal pollution in soil.

## 1. Introduction

Agricultural soils suffer from varying degrees of heavy metal (HM) pollution owing to industrial activities such as mining, metallurgy, and chemical methods, as well as the irrational use of chemical pesticides [1]. The National Soil Pollution Survey Bulletin issued by the Chinese government in 2014 indicated that nearly one-fifth of China’s arable land is contaminated with heavy metals such as Cu, Pb, Zn, and Cd [2]. Chinese soil pollution data for the period 1977–2020 also revealed that the average concentrations of the heavy metals Cu, Pb, and Cd exceeded the screening values for pollution risk set out in the “Soil environmental quality risk control standard for soil contamination of agricultural land” (GB15618-2018) [3]. Agricultural soils in Iran, India, and other countries are contaminated with Cu, Pb, Zn, Cd, and As to varying degrees [4,5]. Heavy metal contamination of soils causes a decrease in the content of photosynthetic pigments and lipid peroxidation in plants, thereby inhibiting plant growth and leading to a decrease in the yield and quality of agricultural products [6,7]. In addition, heavy metal enrichment in edible parts of agricultural products, such as rice or peanut pods, can increase human health risks [8,9]. *Sedum alfredii* Hance, a horticultural plant with notable ornamental value, has a strong tolerance to heavy metals due to its unique vacuolar isolation mechanism, allowing it to grow in soils heavily polluted with heavy metals [10]. As such, cultivating *Sedum alfredii* Hance in soils contaminated with high concentrations of heavy metals offers a promising approach for the safe and effective utilization of polluted agricultural soils.

In situ passivation is a commonly used technology for soil heavy metal pollution control, mainly through the addition of exogenous repair agents to change the form of heavy metals, reduce the available heavy metal content in the soil, and mitigate their toxicity to plants and potential health risks [11]. Commonly used repair agents include clay minerals, metal oxides, biochar, and industrial wastes [12,13,14]. Biochar is a porous, stabilized carbon material produced from waste biomass that has been pyrolyzed under hypoxic conditions. Because of its advantages of low cost, easy availability, and environmental protection, it has attracted widespread attention in the treatment of soil heavy metal pollution [15,16]. BC application reduces the available heavy metal content in the soil and promotes plant growth [17].

Modified BC shows superior performance in managing soil heavy metal pollution. Mg-modified BC can increase the type and number of oxygen-containing functional groups on the surface of BC and increase its adsorption capacity for heavy metals [18], thereby reducing the available heavy metal content in the soil and increasing crop biomass [19,20]. However, during the Mg modification process, MgO enters the BC pores, leading to a decrease in its pore volume and specific surface area, which limits the ability of Mg-modified BC to passivate heavy metals in heavily contaminated soils [21]. Loading natural silicate palygorskite with colloidal properties and rod-like crystal structures onto BC by pyrolysis can greatly increase its specific surface area [22] and adsorption capacity for heavy metals [23]. Therefore, biochar comodified with Mg and palygorskite can more effectively reduce the available heavy metal content in the soil, but its passivation effect on soil heavy metals is affected by the modification method, and studies on its effect on plant growth in heavily polluted soil are lacking.

This study aims to develop Mg-palygorskite-modified biochar to enhance the growth of the horticultural plant *Sedum alfredii* Hance in soils contaminated with Cu, Pb, Zn, and Cd and to explore its underlying mechanisms. Specifically, (1) four types of modified biochar were prepared using MgCl_2_, palygorskite, and sawdust as raw materials through pre-modification and post-modification methods. A greenhouse pot experiment was conducted to investigate plant responses after the application of Mg-palygorskite-modified biochar; (2) by analyzing the physicochemical properties of the modified biochar, the availability of heavy metals and nutrient availability in the rhizosphere soil, the potential mechanisms and key influencing factors of biochar’s effect on plant growth were revealed. The results of this study fill the gap in research on the passivation effects of modified biochar on soil heavy metals and its impact on plant growth, providing fundamental data to support the development of technologies for soil remediation and agricultural management based on modified biochar materials.

## 2. Materials and Methods

### 2.1. Soil Preparation

The experimental soil was topsoil (0–20 cm) from a vegetable farmland in Qingdao City, Shandong Province, China, with a pH of 6.42. The soil samples were dried naturally and ground through a 2 mm nylon sieve after removing stones and other impurities. Four heavy metal solutions of analytically pure CuSO_4_·5H_2_O, (CH_3_COO)_2_Pb·3H_2_O, ZnSO_4_·7H_2_O, and CdSO_4_·8H_2_O were added to the soil with a plastic spray bottle. The amount of Cu^2+^ added to the soil was 800 mg·kg^−1^, that of Pb^2+^ and Zn^2+^ was 1000 mg·kg^−1^, and that of Cd^2+^ was 2 mg·kg^−1^, which was mixed and aged. The soil moisture content was maintained at 60% of the maximum field water-holding capacity during aging. After 1 month of aging, the soil was allowed to dry naturally and ground through a 2 mm nylon sieve for potting experiments.

### 2.2. Preparation and Characterization of Biochar

Both pre-modification and post-modification have been used to prepare modified biochar [24,25]. The mixed wood chips of red oak, elm wood, and Platanus were milled, dried, and passed through a 2 mm sieve. The sieved wood chips were anaerobically pyrolyzed at 600 °C for 4 h to produce raw biochar (BC). The sieved wood chips were immersed in 1.18 mol·L^−1^ MgCl_2_ solution (solid–liquid ratio of 1:10 g/mL), and the supernatant was removed and dried after ultrasonication and standing and then pyrolyzed at 600 °C for 4 h to produce Mg-modified biochar (MBC) prepared via the pre-modified method. After mixing the sieved wood chips with palygorskite at a mass ratio of 5:1 and following the same process as that used for MBC, Mg-palygorskite-modified biochar (MPB) prepared via the pre-modification method was produced. BC was immersed in 1.18 mol·L^−1^ MgCl_2_ solution (solid–liquid ratio of 1:10 g/mL), and the supernatant was removed and dried after ultrasonication and standing and then pyrolyzed for 4 h at 600 °C to produce Mg-modified biochar (BCM) prepared via the post-modification method. After mixing sieved wood chips with palygorskite at a mass ratio of 5:1 and following the same process as for BCM, Mg-palygorskite-modified biochar (BPM) was produced.

The surface morphology and elemental content of the biochar were observed by scanning electron microscopy-energy dispersive spectroscopy (7593-H, Horiba, Montpellier, France) [26]. The mass change curve of the biochar was obtained via a thermal gravimetric analyzer (STA449, Netzsch, Selb, Germany) in an N_2_ atmosphere at a rate of 10 °C/min up to 800 °C [27]. The surface functional groups of the biochar were analyzed via Fourier transform infrared spectroscopy (Tensor 27, Bruker, Karlsruhe, Germany) [28]. The mineral composition of the biochar was analyzed via an X-ray diffractometer (D8 Advance, Bruker, Karlsruhe, Germany) [29]. The ash content was measured by heating the biochar in a muffle furnace at 750 °C for 6 h [30]. The pH and EC were determined via a pH meter and a conductivity meter (solid–liquid ratio of 1:20) [31]. The elemental analyzer (C, H, and N) (Flash2000, Thermo Scientific, Waltham, MA, USA) was used to determine the C, H, and N element content of the biochar. The O content was obtained by the difference calculation method, and the calculation formula was O% = 1 − (C% + N% + H% + ash content%) [32]. The total Mg content (TMg) and water-soluble Mg content of the biochar were determined via inductively coupled plasma–mass spectrometry (ICP-MS) (NexION 350, PerkinElmer, Waltham, MA, USA [33]).

### 2.3. Pot Experiments

*Sedum alfredii* Hance was selected as the test plant because of its strong tolerance to heavy metal stress [34]. A pot experiment was used to study the effects of the above five biochar types on the growth of *Sedum alfredii* Hance seedlings in heavy metal-contaminated soil. The specific steps of the potting experiment were as follows: (1) BC, MBC, MPB, BCM, and BPM were added to the aged soil at 1.5% and 3% (wt%), mixed well and set aside, previous studies have shown that these two dosages can promote plant growth in heavy metal-contaminated soil [35,36]; (2) 500 g of the mixed soil was added to polyethylene pots (12.5 cm × 11 cm × 9.5 cm), and another control without biochar was set up, with a total of 11 treatment groups; (3) *Sedum alfredii* Hance seedlings were transplanted with no obvious difference in plant morphology or quality in each pot and placed in the greenhouse for 49 d of light cultivation; (4) soil moisture was maintained at 60% of the maximum field water holding capacity by weighing and rehydrating the soil regularly every day during the cultivation period, and the position of the pots was adjusted randomly at regular intervals to minimize systematic errors caused by light and temperature.

At the end of the incubation period, plant height, stem diameter, and crown width were measured, and aboveground and underground plant samples and rhizosphere soil samples were collected. The roots were lifted from the soil and gently shaken to collect rhizosphere soil (soil adhering to the roots). The contents of available Cu (ACu), available Pb (APb), available Zn (AZn), and available Cd (ACd) in the rhizosphere soil were determined via ICP–MS after leaching in 0.11 mol·L^−1^ acetic acid solution. The pH and EC of the rhizosphere soil were determined via a pH meter and conductivity meter (solid–liquid ratio of 1:2.5). The NH_4_^+^-N and NO_3_^−^-N contents were determined via a spectrophotometric method with indophenol blue reagent and phenol disulfonic acid, respectively, and the available phosphorus (AP) content was determined via the Mo–Sb Anti-spectrophotometric method. The plants were divided into aboveground and underground root systems, which were rinsed well with distilled water and drained on filter paper, and the fresh masses of the aboveground and underground parts were recorded. Root morphology was analyzed via root scanners (10000XL, Epson Scanning, Suwa, Japan) and WinRHIZO software (WinRHIZO 2005a, Regent, Sherbrooke, QC, Canada). The aboveground and underground parts of the plants were desiccated at 105 °C for 30 min and then dried at 60 °C to a constant mass, and the dry masses of the aboveground and underground parts of the plants were recorded.

### 2.4. Adsorption Experiment

A 0.01 mol·L^−1^ NaNO_3_ solution was used to prepare the Cu^2+^, Pb^2+^, and Zn^2+^ stock solutions at a concentration of 200 mg·L^−1^ and the Cd^2+^ stock solution at a concentration of 50 mg·L^−1^. Considering the difference in the buffering capacity of biochar, NaOH and HNO_3_ (0.01 mol·L^−1^) were used to adjust the pH of the background solution and the heavy metal stock solution so that the pH of the solution system at the time of adsorption equilibrium was 5.0. The above five biochar (50 ± 5 mg) were weighed in 40 mL glass vials, and 35 mL of different concentrations of the heavy metal solutions with the adjusted pH were added to each of the five biochar, which included a total of eight concentration points. Each concentration point was repeated three times, and three groups of blanks (without biochar) were prepared. The solution was shaken at room temperature (25 ± 1 °C, 150 rpm) and protected from light for 36 h. The experiments revealed that the adsorption of the four heavy metals by the biochar reached equilibrium within 36 h. After adsorption, the solution was shaken at 3000 rpm. After shaking, the supernatant was centrifuged at 3000× *g* rpm·min^−1^ for 30 min, and the concentration of heavy metals in the supernatant was determined via ICP–MS analysis. The adsorption isotherms were fitted via the Langmuir and Freundlich models.

(1) $$Q_{e}=\frac{Q_{m}K_{L}C_{e}}{1+K_{L}C_{e}},$$

(2) $$Q_{e}=K_{F}C_{e}^{n}$$

*Q_e_* (mg·g^−1^) and *C_e_* (mg·L^−1^) are the concentrations of adsorbate in the solid and liquid phases at adsorption equilibrium, respectively; *Q_m_* (mg·g^−1^) is the maximum saturated adsorption in the Langmuir model; *K_L_* (L·mg^−1^) is the adsorption coefficient of the Langmuir model; *K_F_* ((mg·g^−1^)(mg·L^−1^)^−*n*^) is the adsorption coefficient of the Freundlich model; and *n* is a nonlinear exponent related to the heterogeneity of the surface location of the adsorbent biochar.

### 2.5. Statistical Analysis

The results of the experiment are expressed as the means ± standard deviations. Excel 2021, R (v4.3.2), and Origin 2023 were used to organize and analyze the data, IBM SPSS software (version 26.0) was used to analyze the significant differences (Duncan test and independent-samples *t*-test), and Jade 6.0 was used to analyze the XRD spectra. A mental test was used to analyze the relationships between biochar properties, rhizosphere soil physicochemical properties, available heavy metal contents, and the morphology and mass of *Sedum alfredii* Hance. The contributions of biochar properties, rhizosphere soil physicochemical properties, and available heavy metal content to the growth effects of *Sedum alfredii* Hance were assessed via structural equation modeling (SEM), a nonsignificant chi-square test (χ^2^/df < 1.5; *p* ≥ 0.05), and a high goodness-of-fit index (GFI ≥ 0.90) to assess the model fitness of SEM [37].

## 3. Results

### 3.1. Properties of Biochar

**Figure 1:**
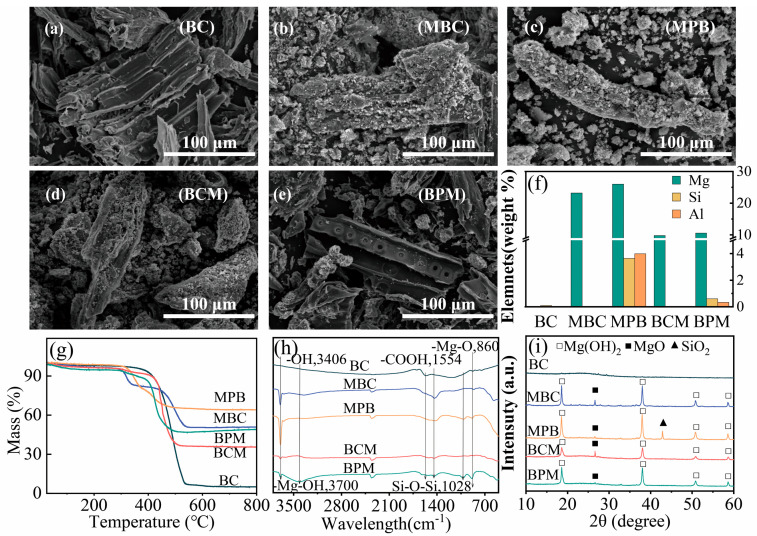
Surface morphology and characteristics of the biochar. SEM images of biochar (**a**–**e**), surface elemental content of biochar determined by EDS (**f**), thermogravimetric analysis of biochar (**g**), FTIR spectra of biochar (**h**), and XRD spectra of biochar (**i**).

As shown in the SEM patterns in Figure 1a–e, the BC had a smooth surface with a tubular fiber structure of woody biochar. Compared with those of BC, the MBC and MPB fiber structures were fragmented with rough and porous surfaces, and MPB had a greater degree of fragmentation. Compared with the pre-modified MBC and MPB, the post-modified BCM and BPM fiber structures were less fragmented but appeared to have more micropores on the surface. The EDS mapping in Figure 1f revealed that the BC surface contained only trace amounts of Si and that the premodified MPB contained relatively high contents of Mg, Si, and Al. Thermogravimetric analysis (Figure 1g) indicated that the mass of all five biochars remained stable above 600 °C, with MPB retaining 64.02% of its original mass, demonstrating the highest thermal stability. The FT-IR spectra (Figure 1h) revealed that the four modified biochar at 864 cm^−1^ for the Mg-O bond [24] and 1554 cm^−1^ for the –COOH bond were not significantly different, and no significant difference in the stretching vibration of the Si–O–Si bond was observed at 1028 cm^−1^ for MPB and BPM [38]. The strongest vibration of the sharp –Mg–OH peak was detected at 3700 cm^−1^ for MPB [39]. The XRD patterns (Figure 1i) revealed that no obvious mineral presence was detected on the BC surface, and characteristic peaks of Mg(OH)_2_ were detected at 18.56°, 37.94°, 50.75°, and 58.57° for the four modified biochar. MgO was detected at 42.85°, and only MPB was detected at 26.62° for SiO_2_.

The physicochemical properties of the biochar are listed in Table 1. The ash content, pH, and EC of the four modified biochar samples were significantly greater than those of the BC samples (*p* < 0.05); the pH of MPB is 10.70, and the ash content is 53.0%, significantly higher than the other three modified biochar samples (*p* < 0.05). With the exception of BCM, the C:N ratio of the other three modified biochar samples was significantly greater than that of the other samples. Except for BCM, the O:C of the three modified biochar significantly increased, by 1.16–2.83 times compared to the original BC, which was conducive to the removal of environmental pollutants [40]. The water-soluble Mg content and total Mg content of the four modified biochar samples were significantly greater than those of the BC samples. These results indicate the successful preparation of modified biochar and that modified biochar has very different properties than BC does.

### 3.2. Effect of Biochar on Morphology and Biomass of Sedum alfredii Hance

**Figure 2:**
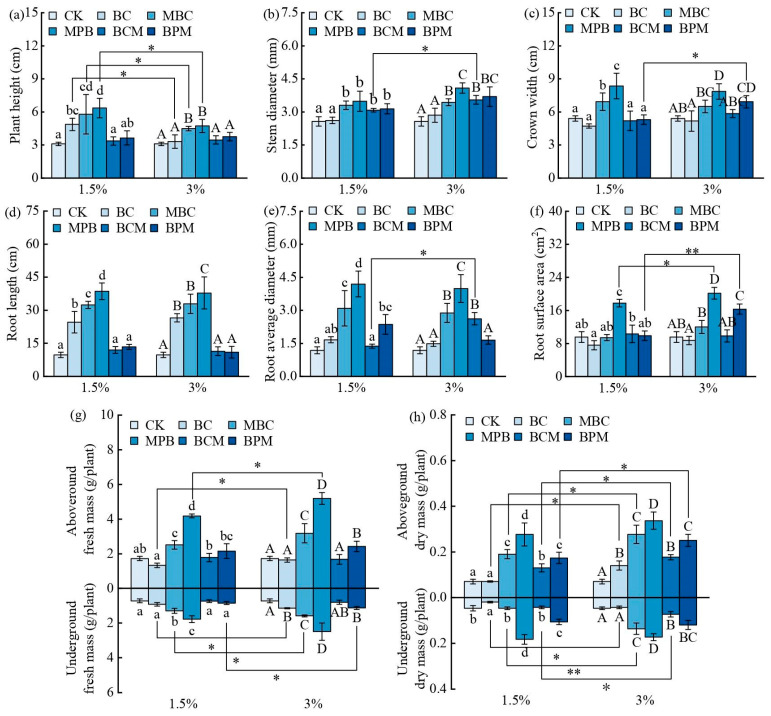
Effects of biochar on the morphology and mass of *Sedum alfredii* Hance. Plant height (**a**), stem diameter (**b**), crown width (**c**), root length (**d**), root surface area (**e**), root diameter (**f**), fresh mass (**g**), and dry mass (**h**) of *Sedum alfredii* Hance under different treatment conditions. CK indicates the control group without biochar addition. Different lowercase letters indicate that there were significant differences in the morphology of *Sedum alfredii* Hance plants among the different biochar treatment groups at 1.5% addition (Duncan test, *p* < 0.05); different capital letters indicate that there were significant differences in the morphology of *Sedum alfredii* Hance plants among the different biochar treatment groups at 3% addition (Duncan test, *p* < 0.05); and asterisks denote significant differences in the morphology of *Sedum alfredii* Hance plants between the treatment groups with different additions of the same biochar (independent samples *t*-test, * and ** represent *p* < 0.05 and *p* < 0.01, respectively); *n* = 3.

Figure 2 shows the effects of biochar on plant morphology and the aboveground and underground mass of *Sedum alredii* Hance. Compared with CK, 1.5% BC significantly increased the plant height and root length (Figure 2a,d), and 3% BC significantly increased the root length, underground fresh mass, and aboveground dry mass of *Sedum alfredii* Hance (Figure 2d,g,h), and 1.5% and 3% MBC and MPB significantly increased the plant height, stem diameter, crown width, root length, average root diameter, and aboveground and underground fresh and dry mass of *Sedum alfredii* Hance. MPB increased more significantly (Figure 2a–e,g,h); 1.5% and 3% BCM significantly increased the stem diameter and aboveground dry mass of *Sedum alfredii* Hance (Figure 2b,h); 1.5% BPM significantly increased the stem diameter, average root diameter, and aboveground and underground dry mass of *Sedum alfredii* Hance (Figure 2b,e,h); and 3% BPM significantly increased the stem diameter, crown width, root surface area, and aboveground and underground fresh and dry mass (Figure 2b,c,f–h). These results indicate that the pre-modified biochar significantly promoted the growth of *Sedum alfredii* Hance, the promotion effect of MPB was more significant, and the post-modified biochar promoted the growth of *Sedum alfredii* Hance to some extent but not as much as the modified biochar did. In soils contaminated with heavy metals, the growth-promoting effect of biochar on *Sedum alfredii* Hance was as follows: MPB > MBC > BPM > BCM > BC.

There were also differences between different additions of the same biochar. Compared with 3% MBC and MPB, a 1.5% addition had more significant effects on plant height, but the other plant morphological parameters and mass showed more significant effects with a 3% addition. For example, compared with 1.5% MPB, 3% MPB significantly increased the root surface area and aboveground fresh mass of *Sedum alfredii* Hance (Figure 2f,g); compared with 1.5% MBC, 3% MBC significantly increased the underground fresh mass and aboveground and underground dry mass of *Sedum alfredii* Hance (Figure 2g,h); compared with 1.5% BCM, 3% BCM significantly increased the stem diameter of *Sedum alfredii* Hance, average root diameter, and aboveground and underground dry mass (Figure 2b,e,h); and 3% BPM significantly increased the crown width, root surface area, underground fresh mass, and aboveground dry mass of *Sedum alfredii* Hance compared with 1.5% BPM (Figure 2c,f–h).

### 3.3. The Effect of Biochar on the Available Heavy Metal Content in Rhizosphere Soil

**Figure 3:**
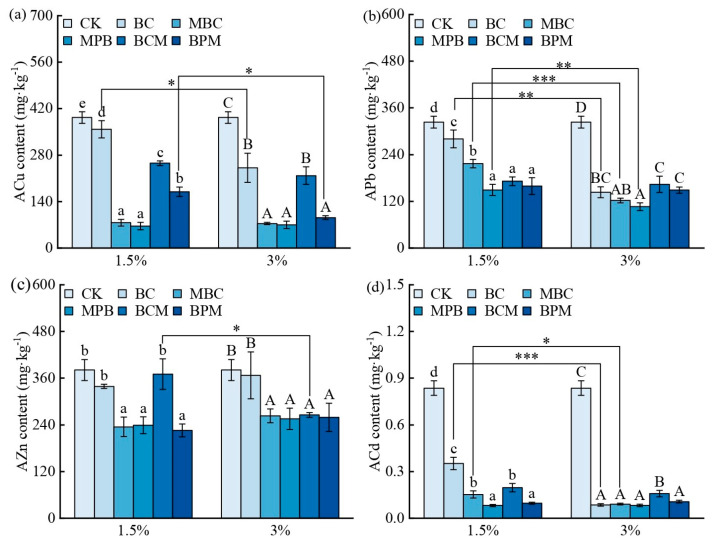
The effect of biochar on the available HM content in rhizosphere soil. Available copper (**a**), lead (**b**), zinc (**c**), and cadmium (**d**) content in the rhizosphere soil of *Sedum alfredii* Hance. CK represents the control group with no biochar addition. Different lowercase letters indicate significant differences in available HM content among different biochar treatment groups at the 1.5% addition rate (Duncan’s test, *p* < 0.05); different uppercase letters indicate significant differences at the 3% addition (Duncan’s test, *p* < 0.05); asterisks denote significant differences in available HM content between different addition rates of the same biochar treatment (independent samples *t*-test, *, **, and *** represent *p* < 0.05, *p* < 0.01, and *p* < 0.001, respectively); *n* = 3.

Previous studies have demonstrated that soil heavy metal contamination inhibits plant growth, resulting in reduced yields and quality of agricultural products [6]. Biochar application can reduce the available heavy metal content and promote plant growth [17,41]. Figure 3 shows the available heavy metal contents in the rhizosphere soils of the different biochar treatment groups. Compared with CK, 1.5% and 3% BC significantly reduced the ACu, APb, and ACd contents in the rhizosphere soil. Compared to BC, 1.5% MBC, MPB, BCM, and BPM significantly reduced the content of ACu, APb, and ACd by 52.7−81.5%, 22.6−46.7%, and 44.2−76.8%, respectively. In addition to BCM, the other three modified biochar significantly reduced the content of AZn by 29.4−33.3%; 3% MBC, MPB, BCM, and BPM significantly reduced the content of AZn by 27.3−30.3%. In addition to BCM, the other three modified biochar significantly reduced the content of ACu by 62.1−71.0%. Overall, the passivation effect of biochar on heavy metals followed the order of MPB > MBC > BPM > BCM > BC, and the effect of 3% biochar addition was greater than that of 1.5% biochar addition. This is consistent with the adsorption characteristics of biochar for heavy metals (Table 2). Moreover, the more significant the passivation effect of biochar on heavy metals is, the lower the available heavy metal content in the soil and the more significant the promotion effect of biochar on the growth of *Sedum alfredii* Hance.

Biochar is a soil heavy metal remediation agent that can mitigate the toxic effects of heavy metals on plants by passivating them to reduce their bioavailability through surface adsorption, ion exchange, co-precipitation, and complexation [42,43]. Table 2 shows the Langmuir and Freundlich adsorption isotherms of Cu^2+^, Pb^2+^, Zn^2+^, and Cd^2+^ on the biochar. The *R*^2^ values indicate that the Langmuir model was superior to the Freundlich model, indicating that the adsorption of the four heavy metals on the surface of the biochar was dominated mainly by monolayer adsorption [44], and the adsorption capacity (*Q_m_*) of the four modified biochar for the four heavy metals was greater than that of the BC, which is consistent with the characteristics of the biochar. Compared with BC, modified biochar has a greater ash content, pH, O/C ratio, and number of oxygen-containing functional groups (Figure 1, Table 1). A high ash content and abundant mineral fractions enhance the ability of these materials to adsorb heavy metals via ion exchange [45]; a high pH enhances their ability to adsorb heavy metals via co-precipitation [25,46], and a high O/C ratio and a rich variety of oxygen-containing functional groups enhance their ability to adsorb heavy metals through complexation [47]. MPB had the highest *Q_m_* in the modified biochar, and its *Q_m_* values for Cu^2+^, Pb^2+^, Zn^2+^, and Cd^2+^ were 1.30, 2.54, 1.32, and 1.25 times greater than those of BC, respectively. Yang et al. prepared MgO modified palygorskite /biochar composite material with a *Q_m_* of 441.1 mg·g^−1^ for Cd^2+^, which is 4.6 times that of the original BC. This study also found that compared to the original BC, Mg-palygorskite-modified biochar has a higher adsorption capacity for heavy metals [18].

### 3.4. The Effect of Biochar on the Physicochemical Properties of Rhizosphere Soil

Biochar improves soil properties and promotes plant growth by increasing the bioavailability of nutrients such as nitrogen and phosphorus [48,49]. Biochar addition affects plant growth by influencing the physicochemical properties of rhizosphere soil (Table 3), which in turn affects the mass growth of *Sedum alfredii* Hance. The addition of modified biochar significantly increased the pH of the rhizosphere soil, which was consistent with the high pH of the modified biochar itself. The modified biochar had little effect on the EC and NH_4_^+^-N contents of the rhizosphere soil but significantly increased the NO_3_^−^-N and AP contents by 30.9−130.8% and 52.1−123.7%.

## 4. Discussion

**Figure 4:**
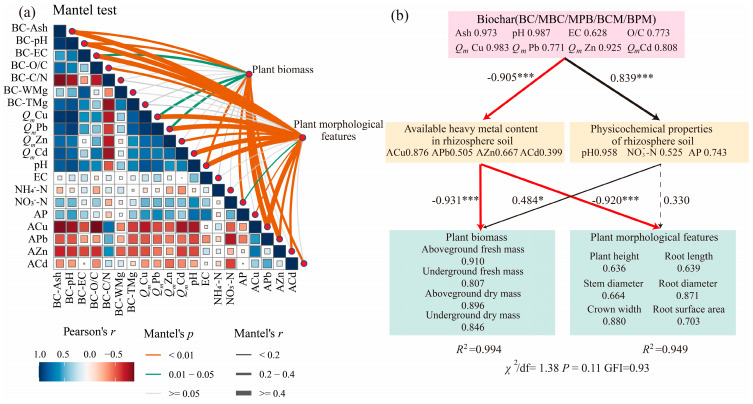
Effects of biochar properties, basic physicochemical properties of rhizosphere soil, and available heavy metal contents on the growth of *Sedum alfredii* Hance. Pairwise Pearson correlation matrix of biochar properties, rhizosphere soil properties, and available heavy metal content (**a**), with the color gradient representing the Pearson correlation coefficient. The relationship between plant growth and biochar properties, available heavy metal content, and rhizosphere soil properties was analyzed using the Mantel test, with line width corresponding to Mantel’s *r* value and line color indicating statistical significance. The structural equation model (**b**) illustrates the effects of biochar addition to heavy metal-contaminated soil on *Sedum alfredii* Hance, showing the influence of various factors. Values on the arrows represent path coefficients (λ), with black and red lines indicating positive and negative correlations, respectively. Asterisks represent statistical significance (* and *** represent *p* < 0.05 and *p* < 0.001, respectively). Solid lines indicate significant relationships, while dashed lines indicate non-significant relationships, and *R²* represents the proportion of variance explained.

The relationships between the biochar properties, physicochemical properties, and available heavy metal contents of the rhizosphere soil and the morphology and biomass of *Sedum alfredii* Hance plants were explored via the Mantel test (Figure 4a). The results revealed that the ash content, pH, EC, *Q_m_*Cu, and *Q_m_*Pb of the biochar and the pH, NO_3_^−^-N, ACu, APb, and ACd contents of the rhizosphere soil were significantly correlated with the mass of *Sedum alfredii* Hance (*p <* 0.05). The ash content, pH of the biochar, and the pH, NO_3_^−^-N, ACu, APb, and ACd contents of the rhizosphere soil were highly significantly correlated (*p <* 0.01) with *Sedum alfredii* Hance mass; the ash content, pH, EC, O/C, and *Q_m_* of the four heavy metals of the biochar and the pH, NO_3_^−^-N, AP, ACu, APb, and AZn in the rhizosphere soil were significantly correlated (*p* < 0.05) with *Sedum alfredii* Hance plant morphology; and the ash content, pH, EC, O/C, and *Q_m_* of the four heavy metals of the biochar and the pH, ACu, APb, and AZn in the rhizosphere soil were highly significantly correlated (*p <* 0.01) with *Sedum alfredii* Hance plant morphology.

In this study, the modified biochar, especially the modified biochar prepared via the premodification method, had different properties from those of BC: the modified biochar had a higher pH, ash content, O/C ratio, richer oxygen-containing functional groups, and mineral content (Figure 1, Table 1). The application of modified biochar, on the one hand, because of its higher pH and ash content (Table 1), can increase the pH (Table 3) of the soil, with heavy metal ions forming insoluble hydroxide precipitates, thus reducing the bioavailability of heavy metals in the soil [48]; on the other hand, modified biochar has a rougher surface structure and richer functional groups and mineral components, which can adsorb heavy metals through a variety of mechanisms (Table 2), such as ion exchange, coprecipitation, and complexation [46,50], which both lead to the fact that after the application of modified biochar, Special MPB, the content of available heavy metals in the rhizosphere soil is significantly reduced (Figure 3), and the content of available heavy metals in the rhizosphere soil directly affects the growth of *Sedum alfredii* Hance (Figure 4). Many studies have shown that biochar reduces the uptake of heavy metals by plants. A meta-analysis revealed that the addition of biochar to soil resulted in average reductions in the concentrations of Cd, Pb, Cu, and Zn in plant tissue by 38%, 39%, 25%, and 17%, respectively [51]. NO_3_^−^-N and AP contents were significantly greater in rhizosphere soils under modified biochar treatments than in those under BC (Table 3), although N (e.g., heterocyclic-N) in biochar is often not available for direct plant uptake and utilization [52]. The readily mineralizable component of biochar accelerates organic matter mineralization and nutrient cycling as well as AMF root colonization [53], and biochar can increase the abundance of P-solubilizing and phosphate-accumulating bacteria, which can increase N and P uptake by plants [49,54]. Modified biochar application reduced the available heavy metal content in the rhizosphere, mitigated heavy metal toxicity, and increased N and P availability, significantly improving the rhizosphere microenvironment and thus promoting the growth of *Sedum alfredii* Hance in heavily heavy metal-contaminated soil.

On the basis of the results of the Mantel test, the indicators with significant correlations with *Sedum alfredii* Hance biomass and plant morphology were selected to construct a structural equation model (Figure 4b), which yielded *p* = 0.11 (*p* > 0.05), GFI = 0.93 (GFI ≥ 0.90), and χ^2^/df = 1.38 (χ^2^/df < 2), indicating that the model was reliable [37]. The results revealed that the available heavy metal content in rhizosphere soil was highly significantly negatively correlated with the biochar content *(p* < 0.001), with a path coefficient of −0.905, indicating that the addition of biochar significantly reduced the available heavy metal content in rhizosphere soil; the mass and plant morphology of *Sedum alfredii* Hance was highly significantly negatively correlated with the available heavy metal content in rhizosphere soil (*p* < 0.001), with path coefficients of −0.931 and −0.920, respectively, which indicated that the lower available heavy metal content in rhizosphere soil was more favorable to the growth of *Sedum alfredii* Hance; the basic physicochemical properties of the rhizosphere soil were highly significantly positively correlated with the biochar characteristics (*p* < 0.001), with path coefficients of 0.839; and the addition of biochar significantly increased the pH, NO_3_^−^-N and AP of the rhizosphere soil. The mass of *Sedum alfredii* Hance was highly significantly negatively correlated with the physicochemical properties of rhizosphere soil, with a significant positive correlation (*p* < 0.05) and a path coefficient of 0.484.

## 5. Conclusions

This is the first attempt to study the effects of Mg-palygorskite-modified biochar prepared in different ways on plant growth in soil contaminated with heavy metals. Biochar addition promoted plant development and increased the mass of *Sedum alfredii*. The growth-promoting effect of biochar on *Sedum alfredii* Hance was as follows: MPB > MBC > BPM > BCM > BC and the promotion effect of 3% biochar addition was more significant. The addition of biochar reduced the available heavy metal content in the rhizosphere soil, and its passivation effect on heavy metals was as follows: MPB > MBC > BPM > BCM > BC. The effect of 3% biochar addition was greater than that of 1.5% biochar addition. Relative to those of BC, the significant increases in ash content, pH, O/C, the number of surface functional groups, and the mineral content of modified biochar, especially pre-modified biochar, were key to the increased adsorption capacity for heavy metals. The results of the Mantel test and structural equation modeling confirm that modified biochar mainly promotes plant development and mass increase in *Sedum alfredii* Hance by reducing the available heavy metal content in rhizosphere soil, increasing the NO_3_^−^-N and AP content in rhizosphere soil.

## Figures and Tables

**Table 1:** Chemical properties of the biochar.

Biochar	Ash (%)	pH	EC (mS·cm^−1^)	Element Ratio		WMg (mg·g^−1^)	TMg (mg·g^−1^)
				C:N	O:C		
BC	0.93 ± 0.31 a	8.75 ± 0.02 a	0.34 ± 0.03 a	19.70 ± 0.18 a	0.19 ± 0.01 a	0.04 ± 0.01 a	1.97 ± 0.43 a
MBC	36.70 ± 3.40 d	10.40 ± 0.02 b	2.84 ± 0.08 d	26.44 ± 1.66 b	0.72 ± 0.06 d	1.25 ± 0.23 bc	122.00 ± 2.70 e
MPB	53.00 ± 0.27 e	10.70 ± 0.03 c	1.81 ± 0.04 b	60.75 ± 5.30 d	0.65 ± 0.04 c	1.05 ± 0.34 b	113.44 ± 3.92 d
BCM	26.10 ± 3.02 b	10.40 ± 0.05 b	2.53 ± 0.07 c	21.38 ± 0.73 a	0.19 ± 0.01 a	1.34 ± 0.12 c	50.80 ± 3.01 b
BPM	31.40 ± 1.44 c	10.40 ± 0.02 b	3.32 ± 0.11 e	38.07 ± 2.24 c	0.40 ± 0.04 b	3.25 ± 0.18 d	81.60 ± 8.32 c

Different lowercase letters in the same column indicate significant differences between biochar for this parameter (*p* < 0.05, *n* = 3).

**Table 2:** The parameters of the HM sorption isotherms of the different biochar samples fitted by the Langmuir and Freundlich models, *N* = 8.

HM	Biochar	Langmuir			Freundlich		
		*Q_m_*(mg·g^−1^*)*	*K_L_*(L·mg^−1^)	*R* ^2^	*K_F_*((mg·g^−1^)(mg·L^−1^)^−*n*^)	*n*	*R* ^2^
Cu^2+^	BC	480.21	0.019	0.990	46.70	0.379	0.918
	MBC	543.53	0.161	0.905	144.39	0.246	0.920
	MPB	625.51	0.121	0.928	151.73	0.259	0.930
	BCM	602.18	0.062	0.908	136.38	0.259	0.884
	BPM	580.22	0.033	0.871	84.89	0.327	0.891
Pb^2+^	BC	344.23	0.024	0.986	38.20	0.366	0.897
	MBC	609.52	0.216	0.934	163.17	0.251	0.920
	MPB	875.82	0.151	0.932	191.78	0.292	0.925
	BCM	782.82	0.737	0.881	237.65	0.239	0.873
	BPM	717.92	0.054	0.939	157.05	0.266	0.900
Zn^2+^	BC	388.19	0.017	0.973	36.56	0.386	0.960
	MBC	410.89	0.058	0.985	82.66	0.280	0.872
	MPB	511.08	0.058	0.972	105.18	0.279	0.942
	BCM	357.19	0.801	0.863	126.36	0.207	0.912
	BPM	351.04	0.056	0.968	64.29	0.300	0.944
Cd^2+^	BC	351.04	0.034	0.989	50.17	0.331	0.926
	MBC	406.43	0.082	0.826	94.39	0.257	0.702
	MPB	440.31	0.537	0.960	169.08	0.178	0.743
	BCM	363.66	0.056	0.937	71.48	0.283	0.864
	BPM	393.01	0.071	0.921	89.12	0.259	0.786

**Table 3:** The effects of biochar on the physicochemical properties of rhizosphere soil.

Addition	Treatment	pH	EC (mS·cm^−1^)	NH_4_^+^-N (mg·kg^−1^)	NO_3_^−^-N (mg·kg^−1^)	AP (mg·kg^−1^)
1.5%	CK	7.05 ± 0.07 a	1.90 ± 0.11 a	18.49 ± 2.51 c	1.39 ± 0.12 a	2.82 ± 0.18 a
	BC	7.23 ± 0.08 a	1.60 ± 0.03 a	14.90 ± 2.06 ab	1.26 ± 0.05 a	3.71 ± 0.30 b
	MBC	8.81 ± 0.20 c	1.82 ± 0.19 a	15.24 ± 1.09 abc	1.89 ± 0.24 b	4.48 ± 0.21 c
	MPB	8.76 ± 0.02 c	1.58 ± 0.12 a	13.66 ± 1.11 a	2.73 ± 0.33 c	4.30 ± 0.37 c
	BCM	8.61 ± 0.15 c	2.35 ± 0.32 b	16.96 ± 1.94 abc	1.99 ± 0.05 b	5.69 ± 0.43 d
	BPM	8.36 ± 0.11 b	1.60 ± 0.07 a	18.19 ± 2.22 bc	1.81 ± 0.15 b	3.19 ± 0.39 ab
3%	CK	7.05 ± 0.07 A	1.90 ± 0.01 A	18.49 ± 2.51 A	1.39 ± 0.12 A	2.82 ± 0.18 A
	BC	7.29 ± 0.04 B	1.90 ± 0.08 A **	16.63 ± 0.35 A	2.40 ± 0.05 C ***	3.39 ± 0.24 A
	MBC	9.51 ± 0.06 F **	2.16 ± 0.11 AB *	15.79 ± 1.87 A	3.20 ± 0.19 E **	5.73 ± 0.16B C **
	MPB	9.35 ± 0.05 E ***	2.16 ± 0.19 AB *	15.25 ± 1.57 A	2.56 ± 0.10 C	6.32 ± 0.57 C **
	BCM	9.08 ± 0.07 D **	2.24 ± 0.11 B	16.72 ± 1.83 A	2.87 ± 0.11 D ***	6.02 ± 0.43 C
	BPM	8.76 ± 0.04 C **	2.12 ± 0.31 AB *	15.23 ± 1.05 A	1.96 ± 0.04 B	5.25 ± 1.40 B **

Notes: CK represents the control group with no biochar addition. Lowercase letters indicate significant differences in the physicochemical properties of the rhizosphere soil of *Sedum alfredii* Hance among different biochar treatment groups at the 1.5% addition rate (Duncan’s test, *p* < 0.05); uppercase letters indicate significant differences at the 3% addition rate (Duncan’s test, *p* < 0.05); asterisks denote significant differences in the physicochemical properties of the rhizosphere soil of *Sedum alfredii* Hance between different biochar addition rates (independent samples *t*-test, *, ** and *** represent *p* < 0.05, *p* < 0.01 and *p* < 0.001, respectively); *n* = 3.

## Data Availability

The original contributions presented in this study are included in the article. Further inquiries can be directed to the corresponding author.
